# Retrospective Analysis of Tuberculosis Treatment Duration and Outcomes Using Explainable Machine Learning on Malaysian Patient Data

**DOI:** 10.3390/jcm15207888

**Published:** 2026-10-13

**Authors:** Vimala Balakrishnan, Jin Khye Tan, Chin Lay Gan

**Affiliations:** 1 Faculty of Computer Science and Information Technology, Universiti Malaya, Kuala Lumpur 50603, Malaysia; 2 Department of Computer Science and Engineering, Korea University, Seoul 02841, Republic of Korea; 3 Centre for Management and Marketing Innovation, CoE for Business Innovation and Communication, Multimedia University, Persiaran Multimedia, Cyberjaya 63100, Selangor, Malaysia; 4 Faculty of Business, Multimedia University, Jalan Ayer Keroh Lama, Melaka 75450, Melaka, Malaysia

**Keywords:** tuberculosis, treatment duration, treatment outcome, machine learning, explainable artificial intelligence

## Abstract

**Background/Objectives**: This study utilizes machine learning (ML) models integrated with explainable artificial intelligence (XAI) to perform a retrospective analysis of factors associated with tuberculosis (TB) treatment duration and outcomes. **Methods**: Two datasets comprising completed TB patient records from a Malaysian hospital were analyzed. Significant features were identified using Pearson correlation for treatment duration and the Boruta algorithm for treatment outcome. Support Vector Regressor (SVR) was used to evaluate treatment duration, while Logistic Regression, Random Forest, and XGBoost were evaluated for treatment outcome classification. SHapley Additive Explanations (SHAP) was integrated to enhance model interpretability. **Results:** On the primary dataset (Dataset A), SVR_Pearson_ demonstrated the best performance for treatment duration (MAE = 44.18 days, R^2^ = 0.54), although performance decreased on the external dataset (Dataset B: MAE = 71.33 days, R^2^ = 0.11), partly due to missing feature imputations. For treatment outcome classification, XGBoost_Boruta_ achieved an accuracy of 99.20% on Dataset A and 97.34% on Dataset B. SHAP feature attribution indicated that treatment outcome was the primary contributor to treatment duration, whereas treatment status and treatment duration were the dominant features contributing to the treatment outcome model. **Conclusions**: These findings demonstrate the value of explainable machine learning for characterizing factors associated with TB treatment duration and outcomes in completed treatment episodes and supporting retrospective clinical audit and post hoc case interpretation.

## 1. Introduction

Tuberculosis (TB) is an airborne infectious disease caused by Mycobacterium tuberculosis, primarily affecting the lungs. It is transmitted through tiny droplets released when an infected person coughs, sneezes, or spits [1]. Despite global efforts to combat TB, it remains a major public health challenge. In 2022, approximately 10.6 million people contracted TB, and 1.3 million people died from the disease globally. In Malaysia, the number of cases rose by 34 cases in 2024, reaching 26,183 cases compared to 26,149 the previous year [2]. The estimated incidence of TB in Malaysia was 96 cases per 100,000 population in 2024 [3].

The current standard treatment for TB involves a six-month regimen comprising a two-month intensive phase followed by a four-month continuation phase [4]. However, this standard treatment approach does not fully account for patient-specific differences in treatment response, leading to varying responses to treatment. Many patients experience treatment failure, relapse, or develop drug-resistant TB, which requires extended, more toxic, and complex regimens. These complications are compounded by disease-related morbidities and comorbid conditions, further emphasizing the need for a better understanding of factors associated with treatment duration and treatment outcomes [5].

TB treatment duration refers to the total length of time a patient undergoes anti-tuberculosis therapy, typically measured from the start to the end of treatment, to ensure successful recovery and prevent drug resistance [6]. In contrast, treatment outcome describes the result of the TB treatment (e.g., cured, failure, etc.). Understanding the factors associated with treatment duration and outcomes can provide insight into variation across completed treatment episodes and support retrospective clinical assessment [7,8]. However, traditional clinical assessments rely on patient-specific risk factors to determine treatment outcomes. Machine learning (ML) approaches can complement conventional analyses by identifying patterns and relationships within clinical data [9]. Recent advancements in artificial intelligence (AI) and machine learning (ML) offer a promising avenue for examining these factors using routinely collected clinical data.

### 1.1. Machine Learning and Tuberculosis

Machine learning (ML) models can process large, complex datasets to uncover patterns and make accurate predictions based on historical information [10]. Previous studies on ML for TB management primarily focused on diagnosis rather than treatment. For instance, Hrizi et al. [11] proposed a hybrid model combining genetic algorithms and Support Vector Machines (SVMs) for TB diagnosis, achieving an accuracy of 84%. In treatment-focused research, Gunasekera et al. [12] developed Logistic Regression models for pediatric TB treatment decisions, achieving high sensitivity (86%) but low specificity (30%).

However, studies focusing on TB treatment duration and outcome are limited. For instance, Ma et al. [9] addressed treatment outcomes in multidrug-resistant TB (MDR-TB) by developing a Logistic Regression model to examine unsuccessful treatment outcomes, using a dataset of 446 cases from Xi’an, China. The model achieved an Area Under Curve (AU) of 0.757 and identified factors such as advanced age and lung involvement as significant factors associated with treatment outcome. Zhang and peers compared a Logistic Regression model with seven ML models using demographic and clinical data to predict TB treatment outcomes for patients with multidrug-resistant or rifampicin-resistant tuberculosis, with results indicating that the Artificial Neural Network (ANN) performed better than the other evaluated models [13]. Similarly, Asad and peers [14] tested various ML algorithms to predict treatment outcomes and found that the ANN achieved the highest accuracy of 78%. Foh et al. [15] tackled class imbalance in TB treatment outcome prediction using Multinomial Naïve Bayes and Synthetic Minority Oversampling Technique (SMOTE), achieving 91.9% accuracy.

Our previous study [16] serves as a baseline for TB treatment duration prediction in this study. The previous study developed an optimized Support Vector Regression (SVR) model, which outperformed Linear Regression, Lasso Regression, Ridge Regression, Random Forest Regression (RFR), and Gradient Boosting Regression (GBR) when estimating TB treatment duration, achieving a Mean Absolute Error (MAE) of 69.73 and Root Mean Squared Error (RMSE) of 109.49. The model was also validated using public TB datasets from Belarus, Nigeria, and Georgia, demonstrating consistent performance across different populations. In the current study, we extend this analysis by addressing outliers in the dataset and evaluating the final model on a new dataset. Additionally, this study develops a model for TB treatment outcome classification and applies explainable AI to examine the contribution of individual features to the model outputs. A web-based interface is also provided to facilitate post hoc exploration and interpretation of completed treatment cases.

### 1.2. Explainable AI and Tuberculosis

**Figure 1:**
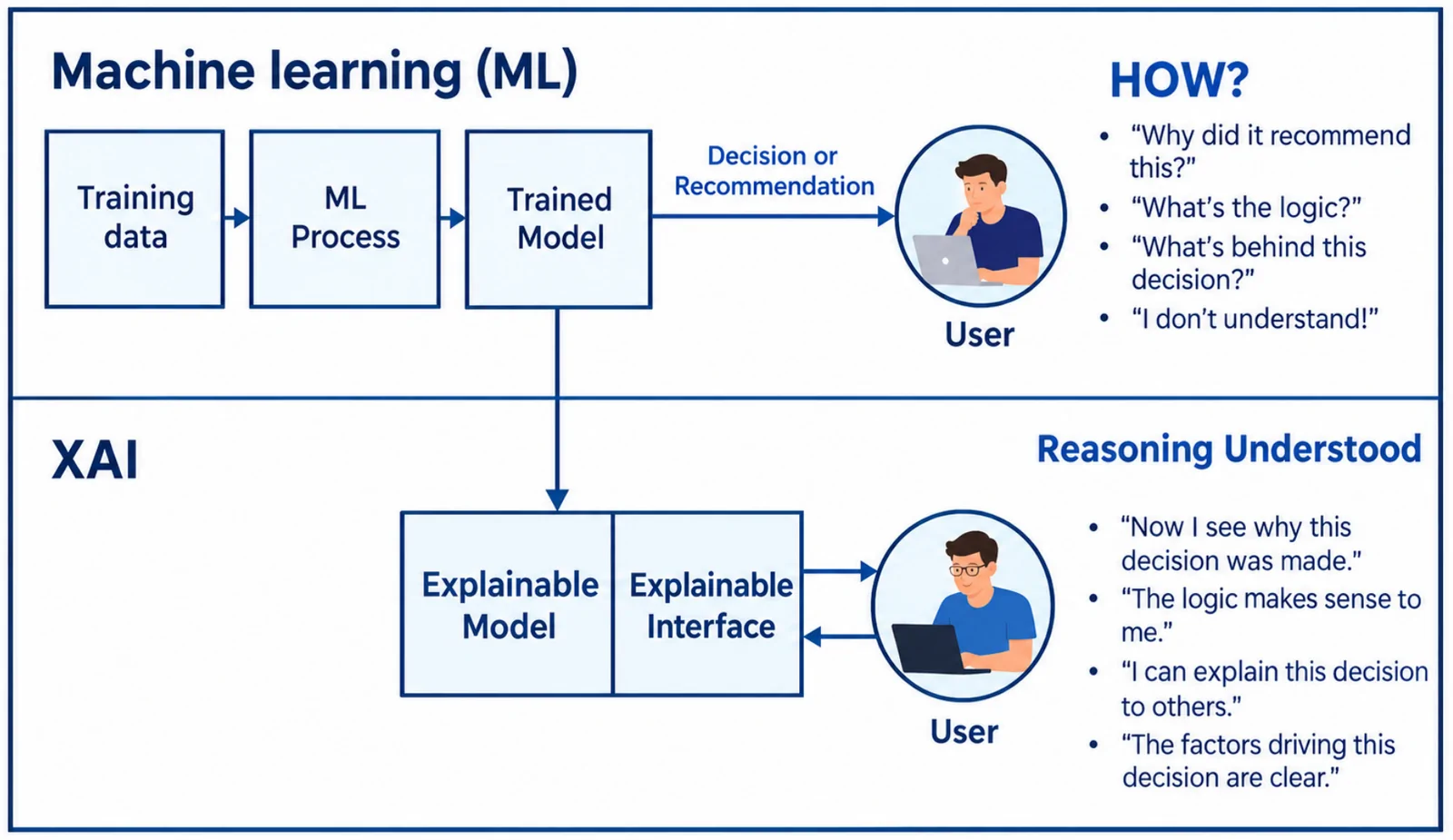
Comparison between machine learning and explainable AI (XAI).

Traditional ML models often function as “black boxes,” offering limited transparency into how predictions are made. This lack of interpretability hinders their adoption in clinical practice, where trust and understanding are paramount. Explainable AI (XAI) overcomes this limitation by providing clear insights into how specific features influence predictions, thereby facilitating informed decision-making [17,18]. Figure 1 illustrates how XAI enhances ML by bridging the gap between opaque recommendations and explainable decisions, helping users understand the reason(s) for predictions.

XAI has been widely used in several domains including finance [19], autonomous vehicles [20], and medical imaging [21]; however, its application in TB remains relatively limited, except for a few recent studies in TB diagnosis [22,23] and treatment outcome prediction [24,25]. For example, Peng and peers [24] developed an XGBoost model for predicting early treatment failure among TB patients with diabetes comorbidities (Area Under Curve (AUC) = 0.9281) and incorporated SHAP to provide clinicians with actionable insights into the model’s predictions. Similarly, authors in [25] interpreted the top-performing ML model’s predictions using SHAP, showing which biomarkers most influenced predictions (e.g., elevated 5′-NT, uric acid, globulin, etc.). On the other hand, Özkurt et al. [22] and Maheswari et al. [23] used SHAP and Local Interpretable Model-agnostic Explanations (LIME) to support XAI in TB diagnosis. Table 1 provides a summary of the related studies, clearly indicating the limited number of studies focusing on TB treatment duration and outcomes, particularly those incorporating XAI.

## 2. Materials and Methods

**Figure 2:**
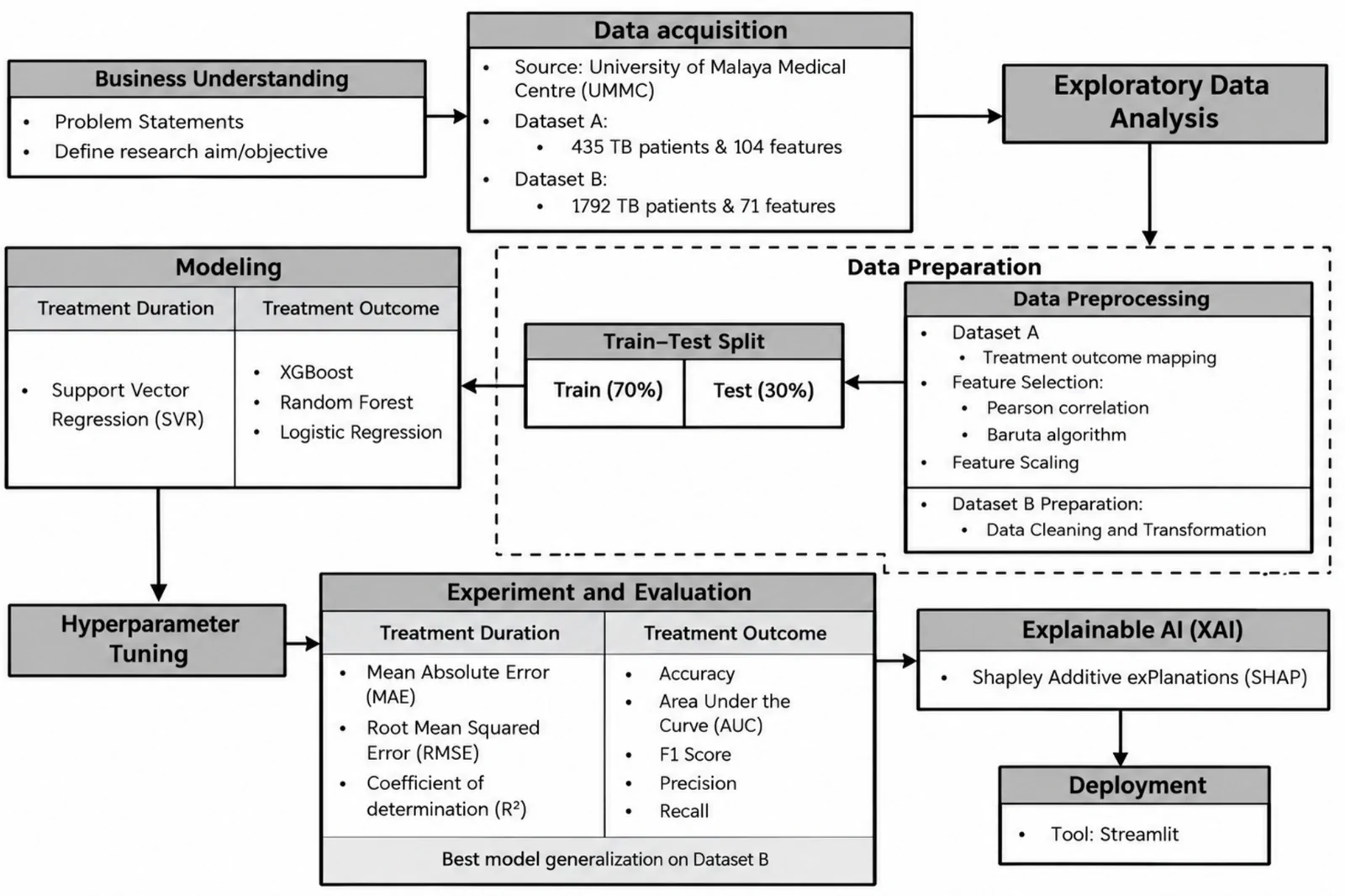
A general overview of the proposed methodology.

The study was based on the Cross-Industry Standard Process for Data Analysis and Predictive Modelling (CRISP-DM) framework [26], which provides a structured approach to data analysis and model development. The overall methodology is illustrated in Figure 2.

### 2.1. Data Acquisition

This study utilized two datasets sourced from the University of Malaya Medical Centre (UMMC) in Kuala Lumpur, Malaysia. The first dataset (herein referred to as Dataset A) was used for training and testing, and the second dataset (i.e., Dataset B) was used to assess the best model’s performance on a new unseen dataset. Dataset A (2019—2020) contained 435 records of TB patients with 104 features. The dataset was also used in our previous study [16], which acts as a baseline in the present study. Dataset B contained TB patients’ data from the same hospital (2021–2023) and serves as a test set to examine the model’s performance on a new, unseen dataset. It contained 1792 records and 71 features. It is worth noting that the number of features was lower than Dataset A (i.e., 71 versus 104); however, the remaining features were the same. Also, there is no overlap of data between the two sets. Supplementary Materials provides the complete details of the datasets used in this study.

### 2.2. Exploratory Data Analysis

#### 2.2.1. Data Pre-Processing

A quality check of Dataset A confirmed it contained 435 records with 104 features, with no missing values or duplicates. Dataset B, initially comprising 1792 records with 71 features, underwent pre-processing to align with Dataset A. First, missing features were identified and imputed using values derived from Dataset A, where numerical variables were replaced with their mean and categorical variables with their mode. Additionally, invalid or incomplete records, such as those with missing essential treatment information or inconsistent start and end dates, were removed. This resulted in a final Dataset B comprising 1201 records with 104 features, ensuring structural consistency between the training and generalization datasets while maintaining data integrity for model evaluation.

The treatment outcome variable in Dataset A was imbalanced, with “Completed” (294) dominating, while “Defaulted” (64), “Died” (72), and “Transferred” (5) were underrepresented. Based on previous studies such as Peng et al. [24], treatment outcomes were recoded into a binary classification task, categorizing “Completed” as successful (1) and all other cases as unsuccessful (0), improving model balance. As for treatment duration, outliers in Dataset A were identified using the Interquartile Range (IQR) method, revealing extreme values at 3962, 436, 439, 445, and 801 days. These were removed to prevent bias in regression models [27]. Feature transformations included standardization using StandardScaler, applied to both Datasets A and B, ensuring numerical variables had a mean of 0 and standard deviation of 1 for models like Support Vector Regression (SVR). Tree-based models such as Random Forest and Gradient Boosting are scale-invariant; hence, they do not require scaling [28].

#### 2.2.2. Feature Selection

Two approaches were used for feature selection, namely Pearson correlation for treatment duration analysis and Boruta for treatment outcome classification. Pearson correlation revealed 41 significant (*p* < 0.005) features for TB treatment duration, with treatment outcome, M_akurit (use of Akurit fixed-dose combination therapy during the maintenance phase; Yes = 1; No = 0) and treatment status (patient status during care, recorded as new, relapse, treatment after default or failure) emerging as the top three features. Contrarily, the Boruta algorithm (i.e., a feature selection method using Random Forest) identified five features for TB treatment outcome classification—weight, maintenance phase regime (MPR), M_akurit, treatment status and duration. The availability of these features varies across the treatment episode: treatment outcome and treatment duration are known at treatment completion, while M_akurit, treatment status, and maintenance phase regime become available at later stages of treatment; therefore, the analysis is intended for retrospective interpretation of completed treatment episodes rather than prediction at treatment initiation. Finally, Dataset A was then split into a 70—30 train–test split for modelling (see Supplementary Materials for the full list of the features).

### 2.3. Modelling

The best-performing model identified in the baseline study, Support Vector Regressor (SVR), was used for treatment duration analysis. SVR is known to model non-linear relationships effectively [27]. As for treatment outcome classification, three models were developed—Logistic Regression, Random Forest, and XGBoost (XGB). These are well-known supervised learning algorithms and have been used in prior studies, as shown in Table 1 [12,24].

### 2.4. Hyperparameter Tuning

Hyperparameter tuning was performed using RandomizedSearchCV with 60 iterations to efficiently explore optimal parameter combinations. Key parameters such as C and gamma for SVR, max_depth and n_estimators for XGBoost, and the number of estimators for Random Forest were adjusted to improve model performance while preventing overfitting.

### 2.5. Experiment and Evaluation

#### 2.5.1. Experiment

All the modelling, experiments, and evaluations were performed using scikit-learn libraries available in Python 3.13, specifically sklearn.ensemblev (https://scikit-learn.org/stable/modules/ensemble.html) sklearn.linear_model (https://scikit-learn.org/stable/modules/linear_model.html), sklearn.svm (https://scikit-learn.org/stable/modules/generated/sklearn.svm.SVC.html), sklearn.model_selection (https://scikit-learn.org/stable/model_selection.html), and sklearn.model_evaluation (https://scikit-learn.org/stable/modules/model_evaluation.html). The experiments were conducted in several scenarios as shown below:

- TB treatment duration analysis:
  - SVR_Default_—model using the 104 original features;
  - SVR_Pearson_—Pearson-selected features (*n* = 41);
  - SVR_Baseline_—baseline features from Balakrishnan et al. [16] (*n* = 8): treatment outcome, treatment status, fixed dose combination dosage, MPR, chest X-ray findings, tuberculin skin test, location of treatment initiation, and levofloxacin-based regimen.
- TB treatment outcome classification:
  - X_Default_—classification model (X) with original 104 features;
  - X_Boruta_—classification model (X) with Boruta features (*n* = 5).

Finally, the best-performing model identified from the experiments above was assessed for its generalizability on Dataset B.

#### 2.5.2. Evaluation

Dataset A was split into 70% training and 30% testing, with 5-fold cross-validation applied during training to improve model stability and reduce overfitting. Key metrics used for treatment duration modelling were MAE, representing the average absolute difference between actual and predicted treatment durations; Root Mean Squared Error (RMSE), which penalizes larger errors by computing the square root of the average squared differences; and R^2^ score, which quantifies the proportion of variance explained by the model, indicating how well the model fits the data [29]. Lower error rates and higher R^2^ scores indicate better model performance.

Evaluation metrics for treatment outcome classification included accuracy (percentage of correctly classified treatment outcomes), precision (fraction of predicted successful treatments that were actually successful), recall (proportion of actual successful treatments that were correctly classified), F1-Score (harmonic mean of precision and recall) and AUC [29]. The higher these scores, the better the classification performance. T-tests were also performed to determine whether the observed differences in model performance are statistically significant [30].

### 2.6. Explainable AI (XAI)

SHapley Additive Explanations (SHAP) was used to generate waterfall plots that visually explain the contribution of individual input features for each patient’s treatment duration and outcome model output. SHAP values attribute importance to each feature by measuring the change in the expected model prediction when conditioning on that feature, providing a unified framework for interpreting model outputs [31,32,33]. Additionally, SHAP calculates feature importance scores, allowing for an in-depth evaluation of which factors most influence the model’s outputs.

### 2.7. Model Deployment

**Figure 3:**
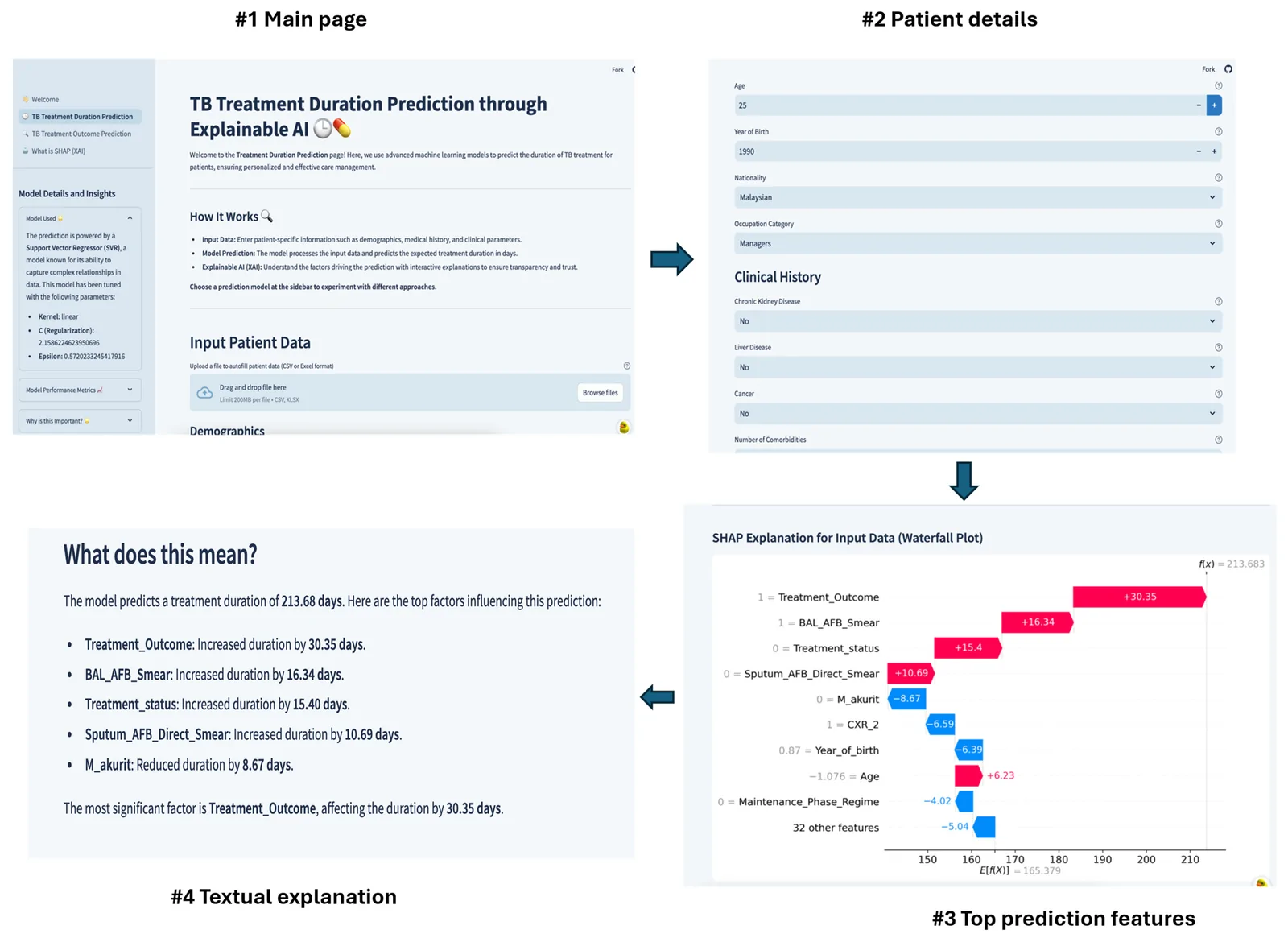
Web-based platform for TB treatment duration.

**Figure 4:**
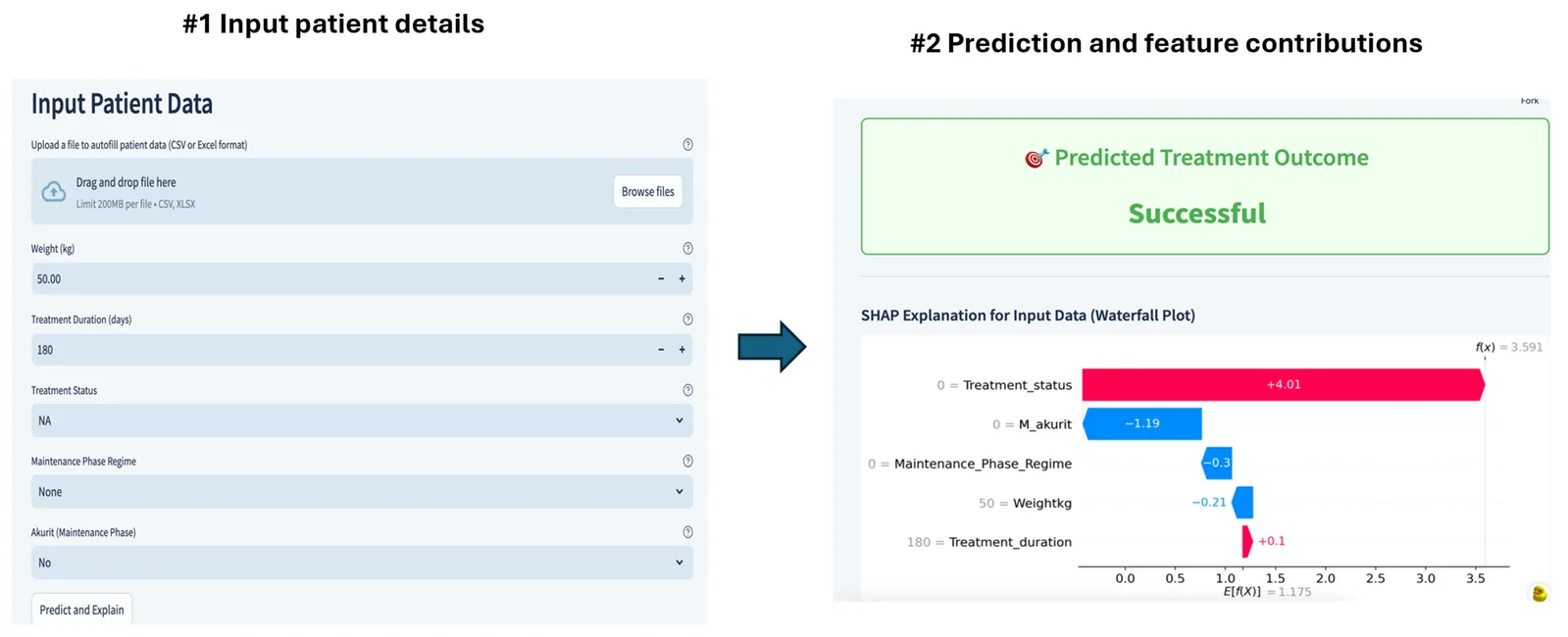
Web-based platform for TB treatment outcome.

Finally, the best models identified from the experiments were implemented in a web-based platform for post hoc exploration of completed treatment cases. Built using Streamlit, the platform provides an interactive interface for visualizing model outputs and SHAP-based feature explanations. Figure 3 depicts an example of TB treatment duration prediction in which a clinician first enters the patient’s data (#1), and the model then predicts treatment duration (i.e., 213.68 days in this example) along with the SHAP feature visualization showing the key features used for the prediction (#2). The platform also includes a more elaborate explanation for the SHAP analysis (#3). Figure 4 shows a similar example for treatment outcome classification.

## 3. Results and Discussion

### 3.1. TB Treatment Duration

Table 2 presents the performance metrics for TB treatment duration analysis on Dataset A for three feature selection scenarios, showing that SVR_Pearson_ yields the best results in terms of a lower MAE and RMSE. The T-test results show the performance of SVR_Pearson_ to be significantly superior compared to SVR_Default_ in terms of the MAE (*p* = 0.028; *t* = 9.27) and RMSE (*p* = 0.003; *t* = 8.13), indicating that treatment duration can be effectively modelled using fewer and more important features, a pattern that was echoed in Balakrishnan et al. [16].

Further, T-tests also revealed the performance difference between SVR_Pearson_ and SVR_Baseline_ to be significant for the MAE (*p* = 0.003; *t* = 11.27) and RMSE (*p* = 0.028; *t* = 9.86). However, the difference in R^2^ (*p* = 0.077) was not statistically significant, suggesting that while the optimized models improved model accuracy, they did not explain significantly more variance in treatment duration. A comparison with Balakrishnan et al. [16], however, indicates a substantial improvement across all the metrics for SVR_Pearson_, with the previous study reporting a higher MAE of 69.73, an RMSE of 109.49, and a much lower R^2^ of 0.36. This could be attributed to the use of more significant features in this study (i.e., 41 versus 8), hence indicating that a greater number of relevant features can improve the modelling of TB treatment duration. Further, although both studies used Pearson to select key features, we believe the performance improvement is probably due to more rigorous data pre-processing in this study, whereby outliers were identified and removed to improve the data quality, hence resulting in more significant features being selected.

### 3.2. TB Treatment Outcome

Table 3 shows the performance of the models for TB treatment outcome classification using default and Boruta-selected features, with results indicating that all the X_Boruta_ models outperformed the X_Default_ models across most of the metrics. However, XGBoost showed consistent improvement across scenarios and outperformed the other models within each scenario. Specifically, XGBoost_Default_ and XGBoost_Boruta_ emerged as the best treatment outcome classifiers compared to the rest of the models, indicating the superior performance of the ensemble classifier compared to other ML models. This is in line with Peng et al. [24], who reported their best treatment outcome classification model to be XGBoost when tested among 429 TB patients in China. A further comparison between these two models revealed XGBoost_Boruta_ to yield better performance results across all the metrics, albeit with no significant difference (*p* > 0.05). Akin to our results for treatment duration, this shows that TB treatment outcomes can be effectively modelled using fewer significant features.

### 3.3. Generalization Performance (Dataset B)

This sub-section provides the best model performance results on Dataset B, that is, SVR_Pearson_ for treatment duration and XGBoost_Boruta_ for treatment outcome. Table 4 provides the results for both datasets for ease of comparison.

It can be observed that the model’s performance dropped substantially and significantly on Dataset B, with the MAE increasing from 44.18 to 71.33 days and R^2^ declining from 0.54 to 0.11. This performance degradation highlights the sensitivity of continuous duration regression to missing clinical variables. We posit this is most probably due to the nature of Dataset B, whereby missing features were artificially imputed using dataset-level means and modes from Dataset A, and thus individual patient variation was flattened. Aggregate statistical imputations can distort feature correlations and reduce model variance explanation in non-linear regression tasks, hence showing that while global imputations maintain structural alignment across datasets, they blur the fine-grained clinical nuances necessary for precise regression modelling [27,28]. These findings indicate that the performance of SVRPearson was affected when applied to Dataset B with imputed features, highlighting the importance of complete and consistent clinical information when interpreting treatment duration models.

Contrarily, XGBoost_Boruta_ showed consistent performance on Dataset B, demonstrating consistent performance across the datasets (Table 5). The Boruta-selected features exist in both datasets; hence, unlike treatment duration, none were imputed. This further supports our explanation above that missing feature imputations could have affected the performance of SVR_Pearson_.

Overall, the results confirm that the models maintain high classification performance when applied to Dataset B, supporting consistent model performance for TB treatment outcome classification across the two datasets.

### 3.4. SHAP Feature Importance

**Figure 5:**
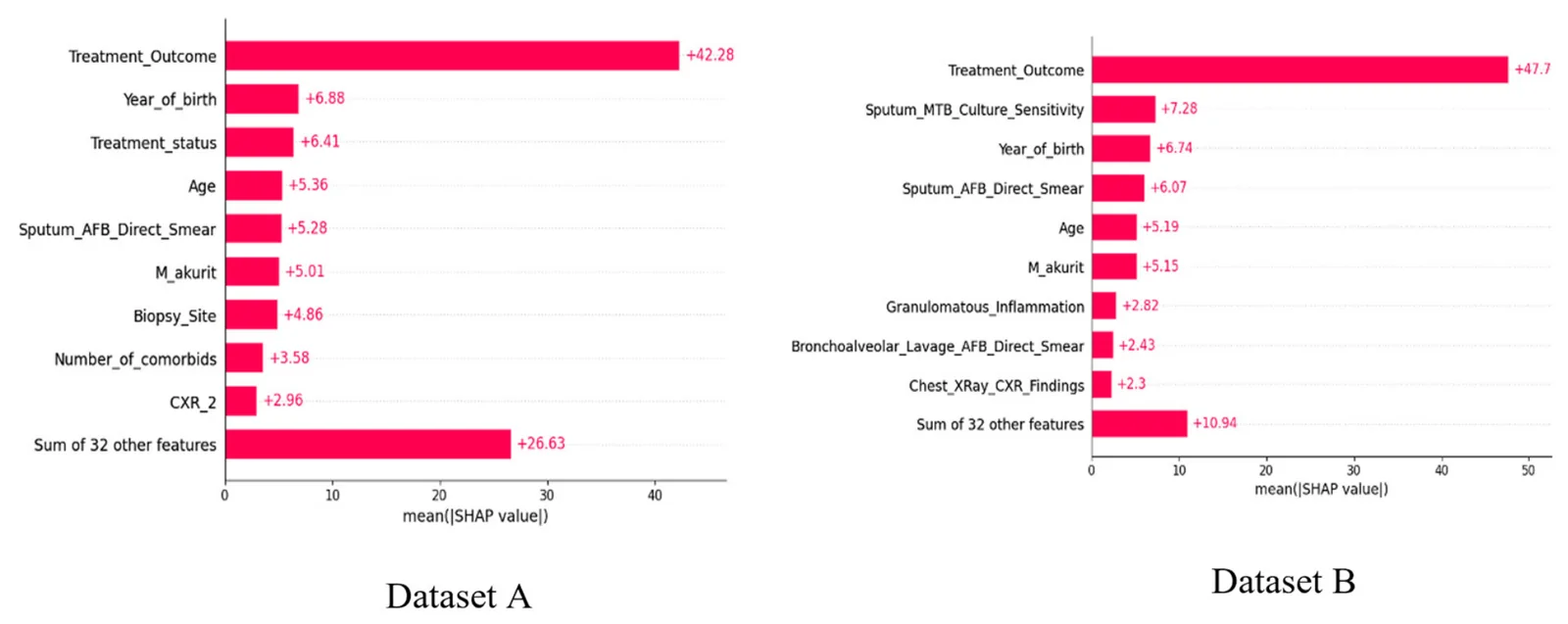
Top features based on SHAP for Datasets A and B for treatment duration analysis. (Note: Sum of 32 features – accumulated score for the rest of the features).

A complementary analysis was performed using SHAP to determine the top nine most influential features for TB treatment duration and treatment outcome based on the selected features from Pearson and Boruta, respectively. This SHAP analysis refers to the top features identified from the 41 and five features selected by the respective techniques used in this study. Looking at the top features for Dataset A and B (Figure 5), treatment outcome was found to have the highest SHAP impact in both datasets (mean score 42.28 and 47.7). It can also be observed that most of the features were consistent, such as year of birth, age, and M_akurit across both datasets.

**Figure 6:**
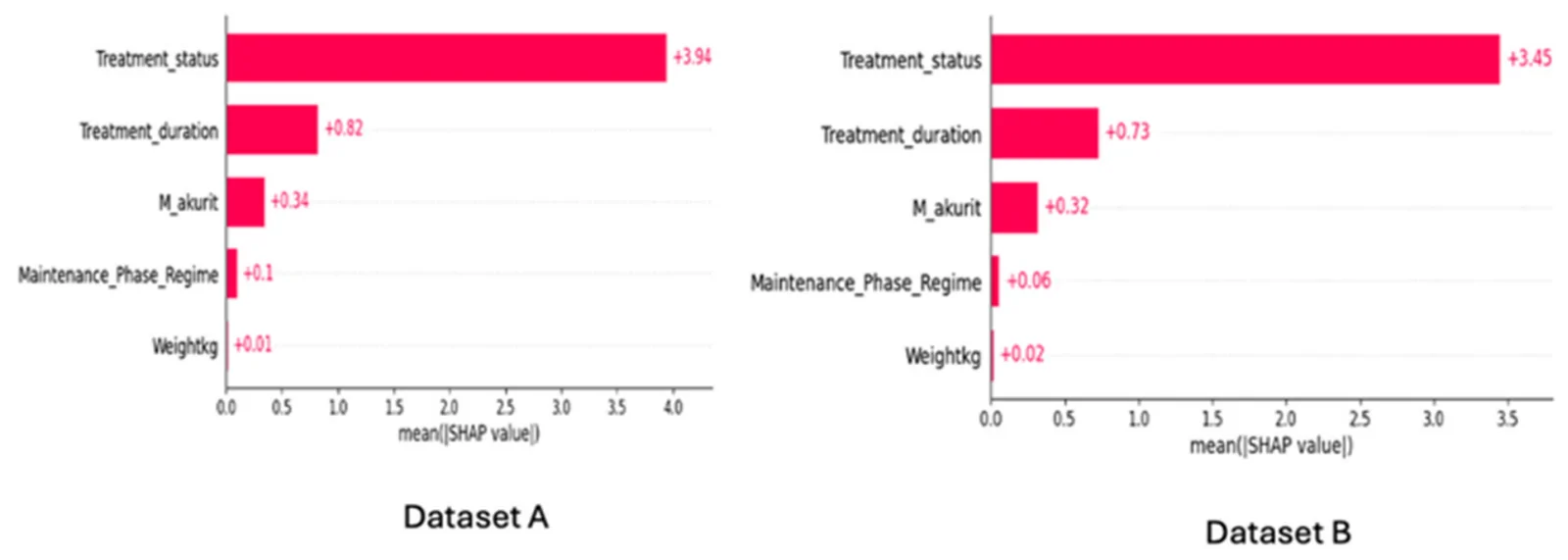
Top features based on SHAP for Datasets A and B for treatment outcome.

The slight variations between Dataset A and B could be due to the imputed features in Dataset B, or there could be other external factors that may influence treatment duration differently across datasets. This highlights the need for multi-method validation when selecting key features for TB treatment duration [34,35]. On the other hand, the top five features for treatment outcome classification for Datasets A and B were consistent, with the emergence of treatment status, duration, M_akurit, MPR and body weight as the most influential features (Figure 6).

This highlights the impact of imputed features and the effect on ML tasks. As shown in this study, model performance and top features were found to be consistent for features that pre-existed in both datasets (i.e., treatment outcome), whilst slight variations in performance and features were observed when some missing features were imputed, as in treatment duration analysis.

## 4. Conclusions, Limitations and Future Directions

This study developed ML models with XAI techniques to retrospectively analyze TB treatment duration and outcomes. Analysis of completed TB patient records revealed SVR_Pearson_ to be the best-performing model for treatment duration, whilst XGBoost_Boruta_ was the best-performing treatment outcome classifier. While XGBoost_Boruta_ demonstrated consistent performance across datasets, SVRPearson experienced a performance drop on the external dataset due to missing feature imputations. Nevertheless, SHAP analysis was instrumental in enhancing model interpretability by identifying key features contributing to model outputs and explaining individual patient outcomes. These findings demonstrate the potential of explainable ML for retrospective analysis and post hoc interpretation of completed TB treatment cases.

While the findings are promising, some limitations warrant consideration. The generalization performance for treatment duration modelling was impacted by the imputed data in the external dataset, which may have introduced variability and reduced model performance. Additionally, the use of data from a single medical centre may limit the generalizability of the findings to broader populations. Therefore, external multicentre validation is essential to assess the consistency of these findings across diverse medical centres, patient populations, and clinical settings. To address these limitations, future studies should incorporate multicentre datasets to ensure diverse representation and develop more sophisticated data imputation techniques for handling incomplete data. Furthermore, exploring hybrid feature selection approaches and integrating additional XAI techniques, such as LIME, could enhance model interpretability.

## Figures and Tables

**Table 1:** Summary of ML studies for TB treatment, TB outcome and XAI.

Study	Focus	Datasets	Algorithms	Findings (Best Model)
[16] (Baseline paper)	Treatment duration	*n* = 435 TB patients from University of Malaya Medical Centre (UMMC)	Support Vector Regression Linear Regression Lasso Regression Ridge Regression Random Forest Regression Gradient Boosting Regression	Optimized SVR MAE = 69.73 RMSE = 109.49
[14]	Treatment Outcome	*n* = 964,099 TB patients from Brazilian Information System for Notifiable Disease (SINAN)	Multinomial Naïve Bayes Synthetic Minority Oversampling Technique (SMOTE)	Naïve Bayes with Borderline SMOTE Accuracy = 91.9% Specificity = 87.6% Sensitivity = 96.1%
[9]	Treatment Outcome	*n* = 446 MDR-PTB patients treated at Xi’an Chest Hospital, China	Lasso Regression Multivariate Logistic Regression Nomogram	Multivariate Logistic Regression AUC = 0.757 C-index = 0.75
[26]	Treatment Outcome	*n* = 2248 TB patients from Chi Mei Medical Center in Taiwan	Multilayer perceptron (MLP) LightGBM Random Forest XGBoost Logistic Regression Support Vector Machine	Mortality prediction best model: MLP with AUC of 0.834. Acute respiratory failure best model: Random Forest with AUC of 0.884. Acute hepatitis best model: XGBoost with AUC of 0.920.
[12]	Treatment Outcome	*n* = 4718 children under 10 years old from 13 studies in 12 high-TB-incidence countries	Multivariable Logistic Regression	Multivariable Logistic Regression Without chest x-ray features: Sensitivity = 86%, Specificity = 30% Without chest x-ray features: Sensitivity = 84%, Specificity = 30%
[24]	Treatment Outcome (treatment failure)	*n* = 429 patients with diabetes from Chongqing Public Health Medical Center	XGBoost Random Forest Support Vector Machine Logistic Regression Boruta algorithm Shapley Additive Explanations (SHAP)	XGBoost AUC = 0.9281 Sensitivity = 71.1% Specificity = 91.7%
[22]	TB Diagnosis	Chest X-ray dataset comprising 700 publicly available TB-positive images and an additional 2800 from NIAID TB portal.	Convolutional Neural Network (CNN) Shapley Additive Explanations (SHAP) Local Interpretable Model-agnostic Explanations (LIME)	CNN F1-Score = 0.94 Precision = 0.93 Recall = 0.95 SHAP gave clearer insights on TB areas, outperforming LIME in clinical interpretation.
[23]	TB Diagnosis	Chest X-ray images from the IEEE Dataport-Tuberculosis database, comprising 1000 images.	Shallow-CNN DenseNet Class Activation Maps (CAMs) Local Interpretable Model-agnostic Explanations (LIME)	Shallow-CNN Accuracy = 0.95 F1-Score = 0.95 CAMs and LIME revealed the Shallow-CNN’s effective focus on TB lung regions, enhancing its clinical interpretability over DenseNet.

Note: The results are provided for the best models only; AUC—Area Under the Curve; MAE—Mean Absolute Error; RMSE—Root Mean Squared Error; MLP—Multilayer Perceptron; CNN—Convolutional Neural Network; CAMs—Class Activation Maps; SHAP—Shapley Additive Explanations; LIME—Local Interpretable Model-agnostic Explanations.

**Table 2:** Model performances for TB treatment duration analysis on Dataset A.

Model	Default (*n* = 104)			Baseline (*n* = 8)			Pearson (*n* = 41)		
	MAE	RMSE	R^2^	MAE	RMSE	R^2^	MAE	RMSE	R^2^
Support Vector Regressor	47.21	65.92	0.54	45.25	66.97	0.52	**44.18**	**63.25**	0.54

Note: MAE—Mean Absolute Error; RMSE—Root Mean Squared Error.

**Table 3:** Model performances for TB treatment outcome prediction on Dataset A.

Models	Default (*n* = 104)					Boruta (*n* = 5)				
	Accuracy	F1	Precision	Recall	AUC	Accuracy	F1	Precision	Recall	AUC
Logistic Regression	0.9435	0.9550	0.9870	0.9242	0.9807	0.9440	0.9560	0.9870	0.9268	0.9821
Random Forest	0.9840	0.9877	0.9878	0.9878	0.9969	0.9840	0.9878	1.000	0.9756	1.000
XGBoost	0.9862	0.9896	0.9949	0.9846	0.9870	0.9920	0.9939	0.9989	0.9878	1.000

Note: accuracy (*p* = 0.0005); F1 (*p* = 0.0007); precision = (*p* = 0.0001); recall = (*p* = 0.0008); AUC = (*p* < 0.0001).

**Table 4:** SVR_Pearson_ performance for TB treatment duration analysis.

Dataset A			Dataset B		
MAE	RMSE	R^2^	MAE	RMSE	R^2^
44.18	63.25	0.54	71.33	145.14	0.11

MAE: *p* = 0.01; RMSE: *p* < 0.001; R^2^: *p* = 0.004.

**Table 5:** XGBoost_Boruta_ performance for TB treatment outcome.

Dataset A					Dataset B				
Accuracy	F1	Precision	Recall	AUC	Accuracy	F1	Precision	Recall	AUC
0.9920	0.9939	0.9989	0.9878	1.000	0.9734	0.9842	0.9727	0.9960	0.9727

Note: All the metrics were significantly different (*p* < 0.001).

## Data Availability

The raw dataset of this study can be obtained from the corresponding author upon reasonable request. Details of the dataset and features are provided in Supplementary Materials, whilst the Python scripts can be found at https://github.com/jinkhye/tb-treatment-xai.

## Supplementary Materials

The following supporting information can be downloaded at: https://www.mdpi.com/article/10.3390/jcm15207888/s1.
