# Supplementary material for: Retrospective Analysis of Tuberculosis Treatment Duration and Outcomes Using Explainable Machine Learning on Malaysian Patient Data
Source: J Clin Med. 2026 Oct 13;15(20):7888. doi: 10.3390/jcm15207888 (PMC13706423; doi:10.3390/jcm15207888)
Supplement: Supplementary file 1 [file jcm-15-07888-s001.zip › jcm-4498705-supplementary.pdf]

Supplementary Materials: Dataset Details and Column Comparisons

This appendix provides a comprehensive description of the datasets utilized in the study for TB treatment duration and outcome prediction, sourced from the University of Malaya Medical Centre (UMMC) in Kuala Lumpur, Malaysia, along with a detailed comparison of columns and features between the old and new datasets.

S.1 Dataset Descriptions

S.1.1 Dataset A: UMMC TB Treatment Data (2019–2020)

- **Source**: University of Malaya Medical Centre (UMMC), Kuala Lumpur, Malaysia.
- **Collection Period**: 1 January 2019 to 30 September 2020.
- **Sample Size**: 435 records.
- **Features**: 104 features, including sociodemographic, clinical, and behavioral variables relevant to TB treatment. Key features include age, gender, nationality, weight, smoking status, BCG scar, diabetes, treatment duration, and treatment outcome.
- **Preprocessing**: The dataset, originally used in Balakrishnan et al. (2023), underwent comprehensive preprocessing, including data imputation, cleaning, and transformation. Missing values were addressed, irrelevant columns were removed, and categorical variables were encoded using label and one-hot encoding techniques. No missing values or duplicates were identified post-preprocessing. Outliers in the treatment duration variable (e.g., 3962, 436, 439, 445, and 801 days) were removed using the Interquartile Range (IQR) method to prevent bias in regression models. The treatment outcome variable, initially imbalanced with 294 "Completed," 64 "Defaulted," 72 "Died," and 5 "Transferred out" cases, was recoded into a binary format: "Successful" (1) for Completed and "Unsuccessful" (0) for all others, enhancing model balance.
- **Purpose**: Used for model training (70%) and testing (30%) via a random split with a fixed random state of 42, ensuring reproducibility. Five-fold cross-validation was applied to evaluate model stability and performance on this dataset.

S.1.2 Dataset B: New UMMC TB Treatment Data (2021–2023)

- **Source**: University of Malaya Medical Centre (UMMC), Kuala Lumpur, Malaysia.
- **Collection Period**: 20 July 2021 to 12 December 2023.
- **Initial Sample Size**: 1,792 records.
- **Initial Features**: 71 features, including similar sociodemographic and clinical variables as Dataset A (e.g., age, gender, nationality, weight, smoking status, BCG scar, treatment outcome).
- **Preprocessing**: Dataset B was raw, containing missing values, null entries, and string-based columns, requiring extensive preprocessing to align with Dataset A's structure. Features were renamed to match Dataset A's schema, and one-hot and label encoding were applied consistently. Missing numerical features (e.g., Weightkg) were imputed with the mean from Dataset A, while categorical features were imputed with the mode. Invalid records (e.g., those with missing or inconsistent start/end dates) were removed, reducing the dataset to 1,201 records. Missing features (31 out of 104)

were added and imputed using Dataset A's mean for numerical variables and mode for categorical variables, resulting in a final dataset with 104 features. Numerical features in both datasets were standardized using StandardScaler to ensure a mean of 0 and standard deviation of 1, except for tree-based models, which are scale-invariant.

- **Purpose**: Used to evaluate the models' generalization ability on new, unseen data, providing insights into their performance on real-world data with imputed values and potential variability.

S.2 Comparison of Columns and Features Between Old and New Datasets

This section provides a detailed comparison of the columns and features between Dataset A (old) and Dataset B (new), including counts of missing and similar columns, as well as missing features identified from Pearson correlation and baseline results.

S.2.1 Summary of Column Comparisons

Category	Count	Description
Missing Columns	31	Columns present in Dataset A but absent in Dataset B.
Similar Columns	79	Columns present in both Dataset A and Dataset B with matching names and roles.
Missing Features (Pearson Correlation)	13	Features identified as missing in the Pearson correlation analysis.
Missing Features (Baseline Results)	3	Features identified as missing in the baseline results analysis.

S.2.2 Detailed Lists of Columns and Features

S.2.2.1 Missing Columns Between Dataset A and Dataset B (n = 31)

- Other_tissuespecimen_collected
- IGRA_test
- Treatment_started_by_which_departmentUnit
- TST_result
- Maintenance_Phase_Regime
- Sputum_smear_clean
- TST
- M_others_specify
- TSPOT_Test
- CXR_2
- Induration
- Intensive_Phase_Regime
- Number_of_comorbids
- HCW
- Type_of_Immunosuppressant

- TB_PCRSeeplexTest
- M_others_dose
- Biopsy_Site
- QuantiFERONTB_Gold_Test
- Biopsy_use
- Cerebrospinal_Fluid_MTB_Culture_SensitivitySensitive
- Case_Detection
- Reason_for_treatment_switch
- BCG_scar_2
- Cerebrospinal_Fluid_AFB_Direct_Smear
- Staging_of_Cancer
- Occupation_category
- Case_detection_2
- Treatment_status
- Treatment_Switch_Regime
- Cerebrospinal_Fluid_MTB_Culture

S.2.2.2 Missing Features from Pearson Correlation Results (n = 13)

- Biopsy_use
- Treatment_status
- TST
- Biopsy_Site
- CXR_2
- BCG_scar_2
- Cerebrospinal_Fluid_AFB_Direct_Smear
- Sputum_smear_clean
- Intensive_Phase_Regime
- Maintenance_Phase_Regime
- Occupation_category
- Number_of_comorbids
- Cerebrospinal_Fluid_MTB_Culture

S.2.2.3 Missing Features from Baseline Results (n = 3)

- Maintenance_Phase_Regime
- Treatment_status
- TST
